# Hybrid Model Structure for Diabetic Retinopathy Classification

**DOI:** 10.1155/2020/8840174

**Published:** 2020-10-13

**Authors:** Hao Liu, Keqiang Yue, Siyi Cheng, Chengming Pan, Jie Sun, Wenjun Li

**Affiliations:** Key Laboratory of RF Circuits and Systems, Ministry of Education, Hangzhou Dianzi University, Hangzhou, Zhejiang, China

## Abstract

Diabetic retinopathy (DR) is one of the most common complications of diabetes and the main cause of blindness. The progression of the disease can be prevented by early diagnosis of DR. Due to differences in the distribution of medical conditions and low labor efficiency, the best time for diagnosis and treatment was missed, which results in impaired vision. Using neural network models to classify and diagnose DR can improve efficiency and reduce costs. In this work, an improved loss function and three hybrid model structures Hybrid-a, Hybrid-f, and Hybrid-c were proposed to improve the performance of DR classification models. EfficientNetB4, EfficientNetB5, NASNetLarge, Xception, and InceptionResNetV2 CNNs were chosen as the basic models. These basic models were trained using enhance cross-entropy loss and cross-entropy loss, respectively. The output of the basic models was used to train the hybrid model structures. Experiments showed that enhance cross-entropy loss can effectively accelerate the training process of the basic models and improve the performance of the models under various evaluation metrics. The proposed hybrid model structures can also improve DR classification performance. Compared with the best-performing results in the basic models, the accuracy of DR classification was improved from 85.44% to 86.34%, the sensitivity was improved from 98.48% to 98.77%, the specificity was improved from 71.82% to 74.76%, the precision was improved from 90.27% to 91.37%, and the F1 score was improved from 93.62% to 93.9% by using hybrid model structures.

## 1. Introduction

Diabetic retinopathy (DR) is an ocular medical disease that damages the retina caused by diabetes. People with diabetes for a longer time are more likely to develop diabetic retinopathy. According to the severity, DR can be divided into the following five grades: no DR, mild, moderate, severe, and proliferative DR. Mild, moderate, and severe are classified as nonproliferative diabetic retinopathy (NPDR) stage. In the NPDR stage, the patients have no obvious symptoms. The way to detect NPDR is to examine the fundus by a trained ophthalmologist. As the condition worsens, DR will develop to Proliferative DR (PDR) stage. In the PDR stage, abnormal new blood vessels form at the back of the eye. These fragile blood vessels can burst and bleed, which blur vision and eventually lead to blindness. So far, the most effective treatment period for DR is in the NPDR stage. Therefore, regular screening of diabetic patients through fundus examination is the most effective method to detect early abnormal signs of DR. Early diagnosis and timely treatment are helpful to prevent DR in patients [1].

However, the screening of diabetic retinopathy needs professional clinical knowledge, experience, and diagnosis time of ophthalmologists. Ophthalmologists generally need to perform a direct examination of the patient's fundus and combine the fundus retinal images taken by special equipment to diagnose the severity of the patient's diabetic retinopathy. This process will take a lot of time. And the number of professional ophthalmologists is far from enough to meet the number of patients diagnosed. Therefore, the automatic classification algorithm of diabetic retinopathy severity plays an important role in improving the efficiency of DR diagnosis. Fundus images, the main images to study DR, are a current research hotspot [2,3]. Some research [4–7] uses machine learning and algorithms for DR detection and classification. However, as deep learning has done well in many competitions, more and more research uses deep learning methods for DR detection and classification. This research mainly focused on the end-to-end DR severity classification of fundus images by using CNNs. In a study, Li et al. [8] presented a novel cross-disease attention network (CANet) to jointly grade DR and DME. They proposed a disease-speciﬁc attention module and a disease-dependent attention module to extract useful features. Their network achieved AUC of 96.3% and accuracy of 92.6% for DR classification on the Messidor database. Shanthi and Sabeenian [9] proposed a modified AlexNet architecture [10] for classification of DR fundus images according to the severity of the disease with the application of suitable Pooling, Softmax, and Rectiﬁed Linear Activation Unit (ReLU) layers to obtain a high level of accuracy. And they validated the performance of the proposed algorithm using the Messidor database [11]. Finally, the proposed algorithm achieved a classification accuracy of 96.6% on the Messidor database. In a study, Hosseinzadeh et al. [12] presented a new feature extraction method using a modified Xception architecture for the diagnosis of DR disease. The proposed method is based on deep layer aggregation that combines multilevel features from different convolutional layers of Xception architecture. The modified Xception architecture that they proposed improved DR classification with a classification accuracy of 83.09% versus 79.59%, sensitivity of 88.24% versus 82.35%, and specificity of 87.00% versus 86.32% when compared with the original Xception architecture. Li et al. [13] extended a baseline network and created four convolutional networks with multiscale inputs. The proposed method obtained a new state-of-the-art kappa score in the task of diabetic retinopathy severity assessment task on EyePACS dataset. In the study [14], Hajabdollahi et al. modified original VGG16-Net [15] to reduce model's structural complexity for DR analysis by a hierarchical pruning method. The proposed method was evaluated using the Messidor database and 35% of the feature maps of VGG16-Net are pruned resulting in only 1.89% accuracy drop. Finally, Jain et al. [16] used 3 different CNN architectures including VGG16, VGG19, and InceptionV3 [17] and evaluated the CNN's performance for 2 classes and 5 classes of DR classification. They found out that the performance of the model was directly linked to the number of convolutional and pooling layers in the CNN. The best accuracy for 2 classes of DR classification was 80.40% achieved by VGG19.

The main contributions of this work are as follows: an improved loss function, enhance cross-entropy (E-CE) loss function, is to improve the performance of basic DR classification models and three proposed hybrid model structures are to fuse multiple basic models for the better performance of DR classification. In this work, preprocessing on the fundus images was firstly performed. During the training process of the basic models, data enhancement methods were used to expand the number of samples and the diversity samples for the DR fundus dataset. And different basic models were trained with E-CE loss and cross-entropy (CE) loss, respectively. Results (see Table 1) showed that our proposed E-CE loss can shorten the convergence time of loss. Under various evaluation metrics, the basic models trained with E-CE loss performed better than the models trained with CE loss. Then, the final output features of the better basic models in different ways were combined to train the hybrid model structures. Results showed that the performance of hybrid model structures is further improved compared to the basic models.

## 2. Materials and Methods

**Figure 1:**
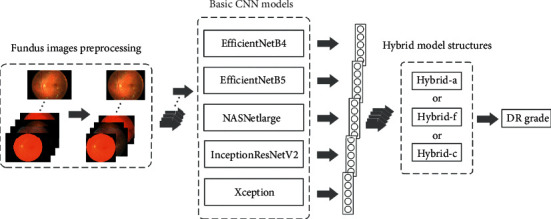
Graph of the proposed algorithm architecture.

The proposed algorithm graph of this work is shown in Figure 1. The graph consists of three steps: fundus images preprocessing, basic CNN models prediction, hybrid model structures prediction, and DR grade output. First, the fundus images would be preprocessed. Then, each basic CNN model predicted the preprocessed fundus images. And the outputs of each basic CNN model were input into the hybrid model structures. Finally, the hybrid model structures output five predicted values, corresponding to the probability of the five DR grades, and the DR grade with the largest probability was taken as the result of the fundus image.

### 2.1. Dataset

**Figure 2:**
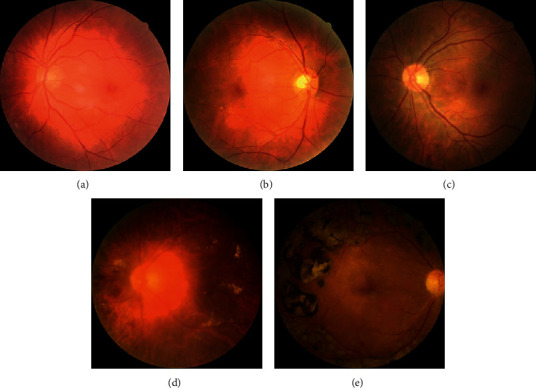
Examples of different severity of DR fundus images. (a) No DR. (b) Mild DR. (c) Moderate DR. (d) Severe DR. (e) Proliferative DR.

The dataset for this work consists of three different datasets which come from the Kaggle diabetic retinopathy detection competition [18] provided by EyePACS, APTOS 2019 Blindness Detection organized by the 4^th^ Asia Pacific Tele-Ophthalmology Society [19], and DeepDR Diabetic Retinopathy Image Dataset provided by the IEEE International Symposium on Biomedical Imaging (ISBI) 2020 [20]. EyePACS dataset contains 35,126 training fundus images and 53,576 test fundus images. APTOS dataset contains 3,662 training fundus images and 1,928 test fundus images. DeepDR dataset contains 1,200 training fundus images, 400 validation fundus images, and 400 test fundus images. All fundus images from the three datasets had been rated for the severity of diabetic retinopathy on a scale of 0 to 4: 0 is no DR, 1 is mild DR, 2 is moderate DR, 3 is severe DR, and 4 is proliferative DR. Examples of different severity of DR fundus images are shown in Figure 2. Each fundus image from the three datasets has a high resolution. The dataset for this work contains 39,988 fundus images which come from the training fundus images with rate of the three datasets because only the training fundus images from the three datasets are rated. As shown in Table 2, the class distribution of the dataset is highly imbalanced, and most of the fundus images are no DR grade.

### 2.2. Data Processing

**Figure 3:**
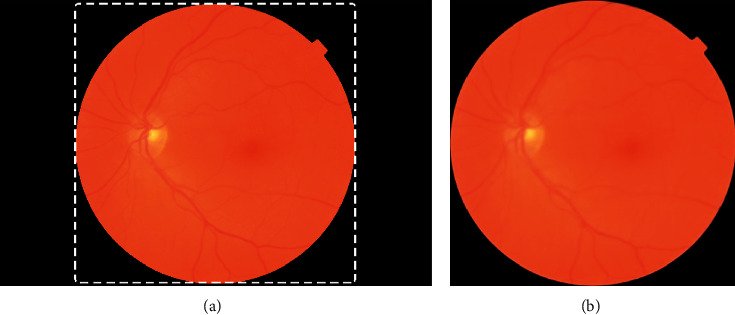
The process of removing the black border of fundus images. (a) The unprocessed fundus image and (b) the processed fundus image.

There are two steps for data processing. One is preprocessing for the fundus images before training basic models; the other is the fundus images enhancement in the training process. The first step for data processing is mainly to remove the black border of the fundus images because the black border will bring useless information and weaken the ability to extract features of the basic models and resize the images to a suitable size for inputs of models. The details are as follows:

1. Binary processing was performed on the fundus images to find the border between the black area and the fundus area and then cut the extra black border for each fundus image. The processes are shown in Figure 3.
2. Because each fundus image has a higher resolution, which is not suitable for the input of the basic models, all images were resized to 380 × 380 pixels for EfficientNetB4, 380 × 380 pixels for EfficientNetB4, 331 × 331 pixels for NASNetLarge, and 299 × 299 pixels for EfficientNetB5, Xception, and Inception-ResNetV2.

In the training process, the following operations were performed on the fundus images: rotation, width shift, height shift, shear range, zoom, horizontal flip, and vertical flip. Then, RandAugment was used for the images. RandAugment [21] is an improved data augmentation method proposed by Cubuk et al. On the ImageNet dataset, Cubuk et al. achieved 85.0% accuracy, 0.6% increase over the previous state-of-the-art, and 1.0% increase over baseline augmentation by using RandAugment.

### 2.3. Basic Model Structures

In this work, hybrid model structures were proposed to improve the classification ability of the basic models. EfficientNetB4, EfficientNetB5, NASNetLarge, Xception, and InceptionResNetV2 CNNs were chosen as the basic models. And then three methods to implement the hybrid model structure were used. Finally, the experiments to verify the performance of the basic models and the basic models with the hybrid model structures were done. The results are shown in part 3. The structure of the basic models are as follows:

1. EfficientNet: EfficientNet [22] is a family of models designed by Tan et al. They proposed a scaling method [23] that uniformly scales all dimensions of depth/width/resolution of CNNs using a simple yet highly effective compound coefficient. Then, they used a neural architecture search to design a new baseline network and used the scaling method to scale it up to obtain EfficientNet, which achieve much better accuracy and efficiency than previous ConvNets. In this work, EfficientNetB4 and EfficientNetB5 were chosen as basic models. The input size of EfficientNetB5 was changed to 299 × 299 pixels and EfficientNetB4 kept the original input resolution. Both of them were added a dropout layer with 0.4 drop rate and a fully-connected layer with 5 units and the activation function of the fully-connected layer was softmax function.
2. NASNetLarge: the NASNet architecture, introduced by Zoph et al. [24], is the best architecture found on CIFAR-10 by the neural architecture search (NAS) framework [25]. Different versions of NASNets with different computational demands can be created by simply varying the number of the convolutional cells and the number of filters in the convolutional cells. The large NASNet-A which performed best on ImageNet image classification was chosen as our basic model. A dropout layer with 0.4 drop rate and a fully-connected layer with 5 units by using softmax function were used to replace the original model output.
3. InceptionResNetV2: InceptionResNetV2 model [26] was proposed by Szegedy et al. InceptionResNetV2 is based on the inception network architecture [27] and replaced the filter concatenation stage with residual connections [28] introduced by He et al. Training with residual connections accelerates the training of inception networks significantly. And residual inception networks outperform similarly expensive inception networks without residual connections by a thin margin. In this work, only the last fully-connected layer with 1000 units was replaced by a fully connected layer with 5 units by using softmax function.
4. Xception: the Xception architecture, introduced by Chollet [29], is a convolutional neural network architecture based entirely on depthwise separable convolution layers inspired by Inception. The Xception architecture has 36 depthwise separable convolutional layers forming the feature extraction base of the network, which makes the architecture very easy to define and modify. The 36 convolutional layers are structured into 14 modules, all of which have linear residual connections around them, except for the first and last modules. In this work, the fully-connected layers and the logistic regression layer of the Xception architecture were replaced by a dropout layer and a fully-connected layer with 5 units by using softmax function.

### 2.4. Hybrid Model Structures

**Figure 4:**
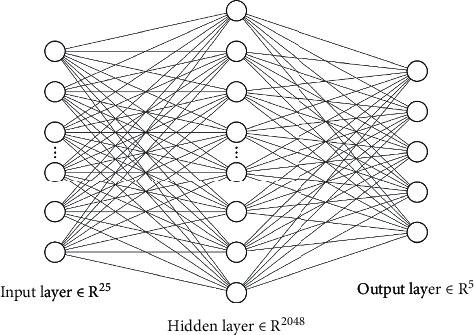
The structure of Hybrid-f.

**Figure 5:**
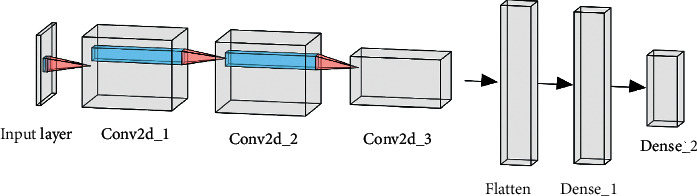
The structure of Hybrid-c.

Three methods were proposed to implement the hybrid model structure, called Hybrid-a, Hybrid-f, and Hybrid-c. The details are as follows:

Hybrid-a: in Hybrid-a, the average value of each DR grade which the basic model outputs is calculated as the final output of the hybrid model structure. The formula is
  (1) $$\begin{array}{c} \begin{array}{c} Y_{\text{grade}}=\frac{1}{N}\sum_{n=1}^{N}y_{n}^{\text{grade}},\;\left(\text{grade}=0,1,2,3,4\right) \end{array}, \end{array}$$
where *N* denotes the number of the basic models. *y*_*n*_^grade^ denotes the DR grade output of the nth model, and *Y*_grade_ denotes the DR grade of the final output of Hybrid-a.
Hybrid-f: Hybrid-f is a model mainly composed of fully-connected layers in short. The output of each basic model, which is a 5 × 1 column vector, is stacked vertically, and finally forms a 25 × 1 column vector as the input of the Hybrid-f model structure.  Figure 4 shows the structure of Hybrid-f. Hybrid-f consists of 2 fully-connected layers. The hidden layer has 2048 units and the output layer has 5 units with softmax activation function.
Hybrid-c: Hybrid-c is mainly composed of 2D convolution layers. The 5 × 1 column vector output of each basic model is stacked horizontally and finally forms a 5 × 5 matrix as the input of the Hybrid-c model structure. The structure of Hybrid-c is shown in Figure 5, and the details of Hybrid-c are shown in Table 3. Three 2D convolution layers as the feature extraction layers make up the first half of the Hybrid-c structure, and then the Hybrid-f structure makes up the last part of Hybrid-c.

### 2.5. Loss Function

Different loss functions have different effects on the training process and results of network models. In this work, an improved loss function, E-CE loss function, was proposed for the training process of the basic models. And comparison experiments with CE loss function were done. The formula of CE loss function is

(2) $$\begin{array}{c} \begin{array}{c} L\left(\hat{y},y\right)=-\frac{1}{N}\sum_{n=1}^{N}\left[y_{n}\log\;{\hat{y}}_{n}+\left(1-y_{n}\right)\log\left(1-{\hat{y}}_{n}\right)\right] \end{array}, \end{array}$$

where *y* denotes the true value, $\hat{y}$ denotes the predicted value, and *N* denotes the total number of DR grade. The E-CE loss function is based on CE loss function and shown as follows:

(3) $$\begin{array}{c} \begin{array}{c} L\left(\hat{y},y\right)=-\frac{1}{N}\sum_{n=1}^{N}\left[y_{n}\log\;{\hat{y}}_{n}+\left(1-y_{n}\right)\log\left(1-{\hat{y}}_{n}\right)+\left|\frac{G_{y}-G_{\hat{y}}}{N-1}\right|\right] \end{array}, \end{array}$$

where *G*_*y*_ denotes the DR grade of truth and $G_{\hat{y}}$ denotes the DR grade of prediction. DR grade is an integer in the range of 0 to 4. In the formula, a part of the loss is added to measure the impact of the misclassification of the basic models during the training process. The farther the output value of the model is from the true value during the model training process, the greater the excess loss will be. Experiments (see Part 3) showed that the E-CE loss function will accelerate the training of the basic models and improve the accuracy of the basic models.

## 3. Results and Discussion

### 3.1. Experiment Setup

Our experiment was carried out on a workstation with 4 NVIDIA GEFORCE RTX-2080Ti GPUs. The memory of each GPU is 11 GB. CPUs are Intel Xeon Silver 4110 processors, 2.1 GHz, a total of 4. The operating system for training models is Ubuntu 16.4. The deep learning framework used in training models is Keras. The backend of Keras used Tensorflow GPU 1.13.1. For each basic training model, the optimizer was RAdam [30] proposed by Liu et al. RAdam, Rectified Adam, is a novel variant of Adam by introducing a term to rectify the variance of the adaptive. And the initial learning rate for each model was set to 0.0008. During the models training, the learning rate could be adjusted automatically. The batch size of EfficientNetB4, EfficientNetB5, NASNetLarge, Xception, and InceptionResNetV2 are 32, 40, 64, 64, and 32, respectively. CE loss function and E-CE loss function were used to train each basic model for the control experiment. The epochs of for training each model were 50. Also, the pretraining weights on the ImageNet dataset were used to accelerate the training process of each basic model. For training Hybrid-f and Hybrid-c model structure, the optimizer was Adam. The initial learning rate was 0.001. And the loss function was cross-entropy loss function. Training epochs were 100.

### 3.2. Performance Evaluation

The performance of the basic models and the hybrid models are evaluated by 5 evaluation metrics which are accuracy, sensitivity, specificity, precision, and F1 score. The formulas are shown as follows, where TP denotes the number of positive samples actually identified as positive samples, TN denotes the number of negative samples correctly identified as the negative samples, FP denotes the number of negative samples falsely identified as the positive samples, and FN denotes the number of positive samples falsely identified as the negative samples:

(4) $$\begin{array}{c} \text{accuracy}=\frac{\text{TP}+\text{TN}}{\text{TP}+\text{TN}+\text{FP}+\text{FN}},\\ \text{sensitivity}=\frac{\text{TP}}{\text{TP}+\text{FN}},\\ \text{specificity}=\frac{\text{TN}}{\text{TN}+\text{FP}},\\ \text{precision}=\frac{\text{TP}}{\text{TP}+\text{FP}},\\ \text{F}1\text{score}=2\cdot \frac{\text{precision}\cdot \text{sensitivity}}{\text{precision}+\text{sensitivity}}. \end{array}$$

### 3.3. Results and Discussion

The basic models were trained on 34,988 fundus images which were selected according to the DR grade ratio from the dataset consisting of EyePACS, APTOS, and DeepDR dataset. The remaining 5,000 images of the dataset were used as test images to evaluate the performance of the models.

**Figure 6:**
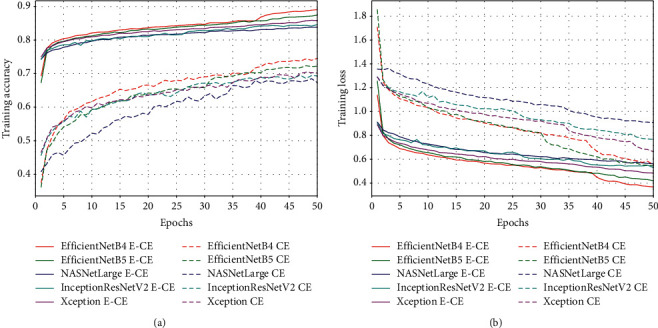
The epochs of training the basic models using E-CE loss and CE loss. (a) Training accuracy varies with epochs for basic models from EfficientNetB4 E-CE to Xception CE. (b) Training loss varies with epochs for basic models from EfficientNetB4 E-CE to Xception CE.

In order to verify the performance of E-CE loss function, each basic model was trained with E-CE loss function and CE loss function, respectively.  Figure 6 shows that the convergence speed of the basic models trained with E-CE loss function is faster than that trained with CE loss function. The accuracy of the basic models is also relatively improved faster.

The accuracy, sensitivity, specificity, precision, and F1 score of the obtained results are shown in Table 1. It can be seen from Table 1 that our proposed E-CE loss function improved the performance of the basic models under partial classification metrics, especially the performance in terms of accuracy and sensitivity. The model trained with E-CE loss function has an average performance improvement of about 5% on accuracy and 3.5% on sensitivity. This may be because an extra part of E-CE loss relative to CE loss increases the influence of the basic models on the misclassification of DR grade during the training process, which will optimize the basic models towards the correct classification faster.

The performance of our proposed hybrid model structures outperforms all the basic models in all classification metrics. Referring to Table 1, Hybrid-c has the highest accuracy which is 0.8634 and F1 score which is 0.939, Hybrid-a has the highest sensitivity which is 0.9877, and Hybrid-f has the highest specificity which is 0.7476 and precision which is 0.9137. As shown in Table 1, in terms of accuracy, Hybrid-c improves EfficientNetB4 by 0.9%, EfficientNetB5 by 1.46%, NASNetLarge by 1.64%, InceptionResNetV2 by 1.32%, and Xception by 1.58%. Results of the experiments prove that the hybrid model structures compared with the single basic model can improve the classification performance in all aspects. The Hybrid-f and Hybrid-c with complex structures have better overall performance than Hybrid-a with simple structure. When the hybrid model structure is more complex, the difference between Hybrid-f and Hybrid-c is smaller. For the hybrid structure proposed in this work, although the higher complexity of the hybrid structure will not bring about a linear performance improvement, the hybrid structure will improve the performance of a single model performance in DR grade classification.

The confusion matrix of Hybrid-c on the testing fundus images is shown in Table 4. From Table 4, Hybrid-c performs the best in DR grade 0 classification, with an accuracy of 0.9706. The performance of Hybrid-c on DR grade 2 classification is better, which achieves 0.7181 score of accuracy. And Hybrid-c has good performance in the classification of DR grade 4, which achieves 0.6698 score of accuracy. For DR grade 1 images, Hybrid-c prefers to misclassify them to DR grade 0. For DR grade 3 images, Hybrid-c prefers to misclassify them to DR Grade 2. The reason for this situation may be as follows:

1. The number of training fundus samples of DR grades 1 and 3 is relatively less compared to the number of DR grades 0 and 2, which causes the poor classification ability of the model for DR grades 1 and 3.
2. The hidden features of fundus images in DR grades 1 and 3 are closer to those of DR grades 0 and 2. We found out the images of DR grades 1 and 3 which were misclassified to DR grades 0 and 2. From the observation of human eyes, the difference between DR grade 1 and DR grade 0 is small, the same as DR grades 3 and 2. For the model, some features extracted by the convolutional layers of DR grade 1 and DR grade 0 may be relatively similar, which may cause some images of DR grade 1 to be misclassified to DR grade 0. This can also explain that for DR grade 4. Although the number of samples in DR grade 4 is small, the features extracted by the model are quite different from those of other DR grades. So, the accuracy of DR grade 4 classification is better than that of DR grades 1 and 3.
3. Experts rating the fundus images may be affected by their own subjective factors and DR grade judgment rules, which may cause some errors in rating DR grades 1 and 3 images. In addition, parts of some fundus images, because of the camera, are dark, blurred, or highlights, which can also affect the judgment of experts.

In future work, we may improve the method of data enhancement to improve the impact of the imbalance of DR grade in the dataset and may extract the output of the intermediate layers of the basic convolution models as the input of the hybrid model structure to increase the richness of the input feature maps of the hybrid model.

## 4. Conclusions

In this work, we proposed an improved loss function, E-CE loss function, and proposed three hybrid model structures Hybrid-a, Hybrid-f, and Hybrid-c to improve the performance of a single model. The results show that the E-CE loss function can effectively accelerate the training process of a single basic model and can improve the performance of a single model compared with the CE loss function. The three different hybrid model structures can improve the performance of the basic models in all aspects. Although the increase in the complexity of the hybrid model does not bring a linear improvement in model performance, the more complex Hybrid-c and Hybrid-f perform better than the simple Hybrid-a in some evaluation metrics. Finally, the proposed algorithm achieved five classifications accuracy of 86.34%, sensitivity of 98.77%, specificity of 74.76%, precision of 91.37%, and F1 score of 93.9% in this work.

## Figures and Tables

**Table 1:** Classification results from the basic models and the hybrid model structures.

		Loss	Accuracy	Sensitivity	Specificity	Precision	F1 score
Basic models	EfficientNetB4	CE	0.8158	0.9442	0.7182	0.9027	0.9230
		E-CE	0.8544	0.9736	0.7061	0.9017	0.9362
	EfficientNetB5	CE	0.7932	0.9254	0.6782	0.8884	0.9065
		E-CE	0.8488	0.9809	0.6549	0.8872	0.9317
	NASNetLarge	CE	0.7828	0.9151	0.7031	0.8951	0.9050
		E-CE	0.8470	0.9845	0.6353	0.8820	0.9304
	InceptionResNetV2	CE	0.7888	0.9657	0.5177	0.8471	0.9025
		E-CE	0.8502	0.9739	0.6963	0.8987	0.9348
	Xception	CE	0.8100	0.9706	0.5742	0.8632	0.9138
		E-CE	0.8476	0.9848	0.6217	0.8781	0.9284

Hybrid model	Hybrid-model-a	CE	0.8584	**0.9877**	0.6481	0.8860	0.9341
	Hybrid-model-f	CE	0.8626	0.9652	**0.7476**	**0.9137**	0.9387
	Hybrid-model-c	CE	**0.8634**	0.9706	0.7325	0.9094	**0.9390**

CE indicates cross-entropy loss function; E-CE indicates enhance cross-entropy loss function; and the bold values indicate the best results.

**Table 2:** The DR grade distribution of the dataset.

Datasets	DR grade					
	0	1	2	3	4	Total number
DeepDR	540	140	234	214	72	1200
APTOS	1805	370	999	193	295	3662
EyePACS	25810	2443	5292	873	708	35126
Total number	28155	2953	6525	1280	1075	39988
Percentage (%)	70.41	7.38	16.32	3.2	2.69	—

**Table 3:** The details of the Hybrid-c structure.

Layer	Units	Filters	Kernel size	Padding	Output shape

Input	—	—	—	—	5 × 5 × 1
Conv2d_1	—	256	3	1	5 × 5 × 256
Conv2d_2	—	256	3	1	5 × 5 × 256
Conv2d_3	—	256	3	0	3 × 3 × 256
Flatten	—	—	—	—	2304
Dense_1	2048	—	—	—	2048
Dense_2	5	—	—	—	5

**Table 4:** The confusion matrix of Hybrid-c.

Predicted DR grade Actual DR grade	0	1	2	3	4
0	3565/97.06	36/0.98	68/1.85	1/0.03	3/0.08
1	204/58.96	87/25.14	54/15.61	0/0	1/0.29
2	140/18.62	47/6.25	540/71.81	15/1.99	10/1.33
3	7/5.69	0/0	55/44.72	54/43.9	7/5.69
4	4/3.78	1/0.94	18/16.98	12/11.32	71/66.98

The first item in each grid cell is the number of fundus images. The second item is the percentage of the images in the DR grade.

## Data Availability

Data used were from the following: Kaggle Diabetic Retinopathy Detection Dataset, available at https://www.kaggle.com/c/diabetic-retinopathy-detection/data; APTOS 2019 Blindness Detection Dataset, available at https://www.kaggle.com/c/aptos2019-blindness-detection/data; DeepDR Diabetic Retinopathy Image Dataset, available at https://isbi.deepdr.org/download.html.
